# Twin Labors

**Published:** 1879-09

**Authors:** 


### TWIN LABORS.

THE following case of twin labor, with both children presenting by an arm, is interesting from the fact of its extreme rarity.

Cazeaux, in his valuable work on Midwifery, does not give an instance of the kind, nor one in which the *first* child assumed this presentation.

In a tabular arrangement of 329 cases of twin labors, he proves, by the absence of such an example, its real interest.

In 329 pregnancies the two children presented as follows:

| | | | |
|---|---|---|---|
| Both by the Head, 134 times. | 1st by Head, 2nd by Breech, 55 times. | Both by Breech, 12 times. | 1st by Breech, 2nd by Head, 31 times. |
| 1st by Breech, 2nd by foot, 11 times. | Both by Feet, 8 times. | 1st by Feet, 2nd by Head, 29 times. | 1st by Breech, 2nd by Elbow, once. |
| 1st by Head, 2nd by Shoulder, 7 times. | 1st by Face, 2nd by Head, once. | 1st by Feet, 2nd by one hand, once. | 1st by Feet, 2nd by Breech, once. |

Many other writers fail to mention cases of this character, and otherwise ignore the subject. In regard to the difficulties encountered in turning the first child, it is almost impossible to find anything in treatises upon midwifery.

LATERAL PLANE PRESENTATION.

Dr. Edwin Borck, in the *Obstetric Gazette*, writes: On the 16th day of March, 1879, at 11 o'clock, P. M., I was requested by Mr. G., to bring my obstetrical instruments and see his wife, living at 1633 Lucas street; age, 30 years; American; brunette; very delicate and nervous; mother of three living boys, aged respectively 13, 6 and 3 years; one boy and one girl dead; never aborted, and had been in labor several hours. Mrs. Granneman, a midwife of Wright street, a very modest and intelligent lady in attendance, who informed me that she had waited upon the patient in her previous labors, that her pains were always very weak and slow, that there had been no progress in this case—pains came regularly, but short and feeble—that there was something wrong, but could not tell what. After tranquilizing the patient by kind words, I found upon vaginal examination that the os was well dilated, membranes protruding and tense; more I could not reach; by manual examination of the abdomen, I felt satisfied that I had to deal with a lateral plane presentation and a very large child. What is the matter? Is anything wrong? were the questions asked.

I gave her one gramme of pot. bromid. to quiet her irritability, console and keep her in good humor, to await the next pain. In a few minutes a very faint pain came on and the membrane ruptured. I hurried to her assistance and found a left hand presenting; in a moment another left hand. I was satisfied now that these were twins, and right and left cephalo-iliac presentation. I pushed the first hand up as far as possible, hunted a leg, which easily came down; the second followed just as easy. Delivery was completed by extension, occiput brachma, face and chin.

Mrs. G. now took charge and cut the cord; after this I saw what was to be done next; putting my left hand upon the abdomen, my right in the vagina, the left hand of child still presenting; again a feeble pain. I pushed upon the arm slightly, a complete spontaneous evolution took place; this child was delivered in vertex presentation—"left occipto-iliac anterior." Mrs. G. now took charge again; she cut the cord and delivered the placenta with ease. No hemorrhage. One of these female children weighed 7½ pounds, the other 6 pounds and 4 ounces; each had its own placenta, but one common membrane.

Mother and offspring did well; nothing but good nursing was required, which the two received in an excellent manner from the hands of the midwife and nurse.

The points of interest are apparent.

St. Louis, Mo., 3613 N. 9th street.

